# The co-distribution of *Plasmodium falciparum *and hookworm among African schoolchildren

**DOI:** 10.1186/1475-2875-5-99

**Published:** 2006-11-03

**Authors:** Simon Brooker, Archie CA Clements, Peter J Hotez, Simon I Hay, Andrew J Tatem, Donald AP Bundy, Robert W Snow

**Affiliations:** 1 Department of Infectious and Tropical Diseases, London School of Hygiene and Tropical Medicine, UK; 2 Schistosomiasis Control Initiative, Imperial College, London, UK; 3 Department of Microbiology and Tropical Medicine, The George Washington University, Washington DC, USA; 4 Spatial Ecology and Epidemiology Research Group, Department of Zoology, University of Oxford, Oxford, UK; 5 Malaria Public Health and Epidemiology Group, Centre for Geographic Medicine. KEMRI/Wellcome Trust Research Laboratories, Nairobi, Kenya; 6 Human Development Division, The World Bank, Washington DC, USA; 7 Centre for Tropical Medicine, University of Oxford, Oxford, UK; 8 Division of Epidemiology and Social Medicine, School of Population Health, University of Queensland, Herston, Queensland, Australia

## Abstract

**Background:**

Surprisingly little is known about the geographical overlap between malaria and other tropical diseases, including helminth infections. This is despite the potential public health importance of co-infection and synergistic opportunities for control.

**Methods:**

Statistical models are presented that predict the large-scale distribution of hookworm in sub-Saharan Africa (SSA), based on the relationship between prevalence of infection among schoolchildren and remotely sensed environmental variables. Using a climate-based spatial model of the transmission potential for *Plasmodium falciparum *malaria, adjusted for urbanization, the spatial congruence of populations at coincident risk of infection is determined.

**Results:**

The model of hookworm indicates that the infection is widespread throughout Africa and that, of the 179.3 million school-aged children who live on the continent, 50.0 (95% CI: 48.9–51.1) million (27.9% of total population) are infected with hookworm and 45.1 (95% CI: 43.9–46) million are estimated to be at risk of coincident infection.

**Conclusion:**

Malaria and hookworm infection are widespread throughout SSA and over a quarter of school-aged children in sub-Saharan Africa appear to be at risk of coincident infection and thus at enhanced risk of clinical disease. The results suggest that the control of parasitic helminths and of malaria in school children could be viewed as essential co-contributors to promoting the health of schoolchildren.

## Background

The economically developing world, particularly sub-Saharan Africa (SSA), bears the brunt of premature mortality, morbidity and disability. Much of this disease burden is the result of endemic parasitic infections that have adapted to tropical ecosystems and their vectors [1]. Among the parasitic diseases, *Plasmodium falciparum *malaria inflicts the largest burden [1,2] and hookworm infection is amongst the most common of all chronic infections, with a third of the continent's population infected at any one time [3,4]. The high prevalence of both infections among individuals living in Africa means that co-infection with *P. falciparum *and hookworm is extremely common [5,6]. There is increasing evidence that co-infection with multiple parasites may impair the immune response of the host to single parasites, and might increase susceptibility to clinical disease in ways that are not at present clearly understood [7-9]. Co-infection with *P. falciparum *and hookworm may also enhance severity of anaemia because of the distinct mechanisms through which each parasite causes anaemia. Finally, there are increasing calls to improve the coordination of national control programmes working to prevent different parasites associated with child mortality and morbidity [10,11]. Despite its potential public health importance and synergistic opportunities for control, there have been no efforts to map the co-distribution of these parasites in Africa.

Geographical distributions of parasitic diseases are increasingly being defined by combining limited geo-referenced disease data with extensive environmental information derived from Earth orbiting satellites [12,13]. The population dynamics of vectors or the free-living parasite forms depend critically upon elements of the weather that can be measured using remotely sensed correlates of rainfall, temperature and land-use [14,15]. Epidemiological and demographic models can be used to relate these data to estimate the distribution of humans and parasites at high spatial resolution [14,16]. Such models can in turn help provide an empirical basis for defining the disease burden of polyparasitism and the potential health impact of removing or reducing disease risk.

Given that hookworm and other nematode species infections are most intense among children aged 5–14 years, the principal focus of helminth control has been among schoolchildren [17]. Within this age range, children in stable transmission areas of Africa are frequently infected with *P. falciparum *[18], and as such, are particularly vulnerable to potential consequences of co-infection. The aim of the present analysis is to develop a predictive map of hookworm in Africa and to define the coincidental geographical distributions of African school-aged populations exposed to *P. falciparum *and to hookworm.

## Methods

### Modelling hookworm distributions

Epidemiological data on prevalence of hookworm were obtained from standardized school-based surveys conducted across SSA during the period 1985–2004. The details and sources of the survey data are provided in Additional File 1. A total of 71,681 children from 1,144 locations were included in the analysis. These data were spatially linked to satellite-derived estimates of land surface temperature (LST) and normalized difference vegetation index (NDVI) for 1982–2000 [19] to investigate the large-scale ecological correlates of infection prevalence. The urban layer of the Global Rural-Urban Mapping Project (GRUMP) [20], which utilizes data on night-time lights and Landsat satellite sensor imagery, in combination with other geographic data, was used to investigate patterns of hookworm prevalence according to urban, peri-urban and rural locations.

Models of predicted prevalence were based on binomial logistic regression analysis, including the school as a random effect. For each location, the response variable contained the total number of positive responses and the number examined, and the independent variables LST and NDVI. Due to non-linear relationships between observed prevalence and predictor variables, the predictors were categorized before being entered into the models. Initial analysis indicated no systematic difference in prevalence according to the degree of urbanization and therefore this variable was not included in the final model. Tests were made for significant interactions between environmental variables and none were found to be significant. The coefficients from the final models were then applied to the categories of the predictor variables to generate a predicted prevalence of infection. Ninety-five percent confidence intervals (CI) were calculated for the logit (predicted prevalence) and the final confidence intervals and predicted prevalence were obtained by transforming them using the "expit" function. Analysis was done using Stata 9 (Stata Corporation, College Station, Texas, USA).

Maps were then created using ArcView version 3.2 (Environmental Systems Research Institute Inc., CA, USA), including surfaces of the lower 95% CI, the mean and the upper 95% CI for predicted risk. Models were then cross-validated using a jack-knifing procedure whereby a single school was excluded, and a logistic regression model was fitted to the remaining data. The coefficients from this model were then applied to the values of the predictor variables from the missing school to generate a predicted probability of occurrence of prevalence >5% (indicative of endemicity) and >50% (WHO defined threshold for the need for mass treatment). The process was repeated for each school. Predicted and observed datasets were therefore independent because the prediction for each school was generated by using prevalence data only from other schools. Predictions were compared to observed values to calculate sensitivity, specificity, positive predictive value and negative predictive value. Receiver operating characteristics (ROC) analysis was also used to calculate the area under the receiver-operator curve (AUC), which provides a composite measure of overall model performance [21].

### Urban corrected malaria risk distribution

Malaria risk is defined on the basis on an existing model which describes climatic conditions that range from unsuitable (0) to completely suitable (1) for stable *P. falciparum *transmission [22,23] as no other endemicity-specific malaria map currently exists for Africa. The Fuzzy Climate Suitability (FCS) index is defined by a series of curves:

y=cos⁡2[(x−U)(S−U)∗π2]
 MathType@MTEF@5@5@+=feaafiart1ev1aaatCvAUfKttLearuWrP9MDH5MBPbIqV92AaeXatLxBI9gBaebbnrfifHhDYfgasaacH8akY=wiFfYdH8Gipec8Eeeu0xXdbba9frFj0=OqFfea0dXdd9vqai=hGuQ8kuc9pgc9s8qqaq=dirpe0xb9q8qiLsFr0=vr0=vr0dc8meaabaqaciaacaGaaeqabaqabeGadaaakeaacqWG5bqEcqGH9aqpcyGGJbWycqGGVbWBcqGGZbWCdaahaaWcbeqaaiabikdaYaaakmaadmaabaWaaSGaaeaadaqadaqaaiabdIha4jabgkHiTiabdwfavbGaayjkaiaawMcaaaqaamaabmaabaGaem4uamLaeyOeI0IaemyvaufacaGLOaGaayzkaaaaaiabgEHiQmaalaaabaacciGae8hWdahabaGaeGOmaidaaaGaay5waiaaw2faaaaa@442D@

where x is a climate parameter, U is the value of x when conditions are unsuitable, and S is the value of x when conditions are suitable. When S is greater than U the suitability (1-y) increases with x; when S is less than U the suitability 1-y decreases as x increases. The model defines monthly increasing curve (S = 22°C, U = 18°C) and decreasing curve (S = 22°C, U = 32°C) for mean diurnal air temperature, a monthly increasing curve (S = 80 mm, U = 0 mm) for rainfall, and a single increasing curve (S = 6°C, U = 4°C) for annual minimum temperature. This model is the most widely used malaria suitability transmission model for Africa, and has become the *de facto *standard for defining population at risk (PAR) for malaria morbidity and mortality estimates in Africa [24-26]. The analysis adopted a classification used in previous estimations of childhood populations at risk of different transmission conditions [27,28]: Class 0 zero risk (FCS = 0), Class 1 marginal risk (FCS >0–<0.25), Class 2 acute seasonal transmission (FCS >0.25–<0.75) and Class 3 stable endemic transmission (FCS >0.75). The FCS risk classes were adjusted for the suppressive effects of urbanization on malaria transmission by identifying population density decay functions associated with urban agglomerations of more than one million persons [26]. The characteristic population densities of urban, peri-urban and rural were then used to create a continuous urban-rural surface for the continent by applying these thresholds to the human population distribution map (see below) and calculating the reduction in malaria transmission, as measured by the entomological inoculation rates characteristic of these land-use classes [26].

### Estimated populations at risk

Population data are derived from the Gridded Population of the World (GPW) version 3.0 [20]. GPW3.0 is a global human population distribution map derived from areal weighting of census data from 364,111 administrative units to a 2.5' × 2.5' spatial resolution grid. Each grid cell represents the residential population count for the year 2000. Country-specific medium variant population growth rates and proportions of the population aged 5–14 years from the United Nations Population Division – World Population Prospects (UNPD-WPP) database [27] were used to project this age cohort of the population total to 2005 using Idrisi Kilimanjaro (Clark Labs, Clark University, MA). This population denominator surface was then used to derive population at risk estimates. Extractions of population at risk by prevalence of hookworm and by malaria endemicity class were then conducted in ArcView 3.2. To estimate the combined risk of hookworm and *P. falciparum*, it was assumed that there was independence between species. Since urbanization was not found to be significant in patterns of hookworm infection no form of urban correction was undertaken for hookworm. However, the suppressive effects on *P. falciparum *transmission have been well documented [26,28] and thus populations exposed to various malaria transmission intensities were adjusted as described above.

## Results

**Figure 1:**
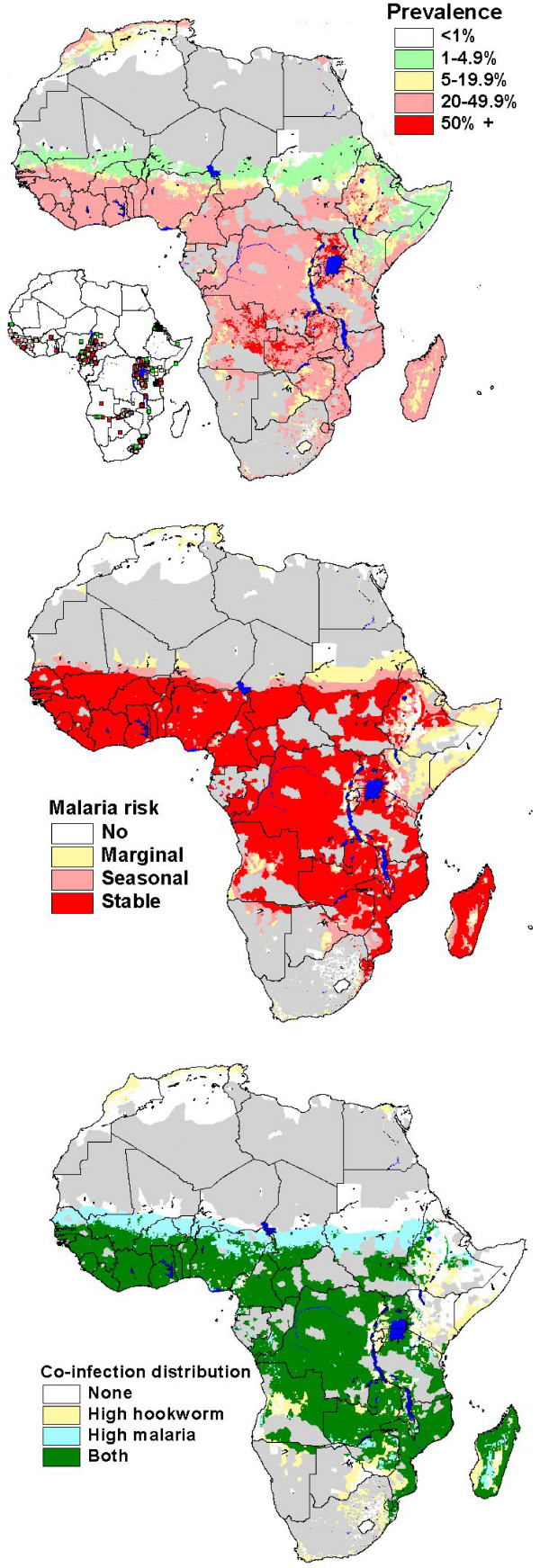
(top) Predicted prevalence of hookworm, based on relationships between observed prevalence of infection among school-aged children (insert) and satellite-derived environmental data; (middle) map of climatic suitability for *P. falciparum *malaria transmission based on Craig et al. (24), adjusted for urbanization (28); and (bottom) map of geographic overlap of moderate-high hookworm (prevalence >20%) and *P. falciparum *transmission. Grey indicates population density <1 km^2^.

Table 1 presents the binomial logistic regression model of the prevalence of hookworm and shows that prevalence was maximal at temperatures between 32–45°C and at elevations of 1000–1500 m, and was positively associated with NDVI. Cross-validation using the jack-knifing procedure indicated that the model had reasonable predictive accuracy, as indicated by the AUC statistic and estimated sensitivity and specificity (Table 2). The model was subsequently used to develop a predictive map of hookworm prevalence (Figure 1a). This shows that infection is widely distributed across SSA. Several countries show minimal levels of infection (*e.g*. Eritrea, Mali, Mauritania and Niger) where high temperatures and limited moisture limit parasite transmission. In many of these areas, the population density is less than one per square kilometre. The prevalence of hookworm infection is spatially heterogeneous in Botswana, Chad, Ethiopia, Namibia, Senegal, Somalia and Sudan. For the remaining countries, most of the population is exposed to moderate-high levels of hookworm infection. It is estimated that of the 179.3 million school-aged children 5–14 years who live in SSA, 50.0 (95% CI: 48.9–51.1) million) (27.9% of total population) are infected with hookworm.

**Table 1:** Binomial logistic regression model of the prevalence of hookworm in sub-Saharan Africa

Variable	OR	95%CI	p-value
LST			
<29°C	1.00		
29 – 32°C	1.02	0.94 – 1.11	0.624
32 – 37.5°C	2.16	1.99 – 2.34	0.000
37.5 – 45°C	2.49	2.27 – 2.72	0.000
>45°C	0.93	0.80 – 1.07	0.308
Elevation			
<500 m	1.00		
500 – 1000 m	0.60	0.58 – 0.63	0.000
1000 – 1500 m	1.46	1.41 – 1.52	0.000
>1500 m	0.50	0.46 – 0.55	0.000
NDVI			
<-7.8	1.00		
-7.8 – -6	3.56	3.07 – 4.14	0.000
-6 – -5	6.73	5.77 – 7.85	0.000
>-5	10.55	9.01 – 12.35	0.000

Logit estimates, Number of obs = 71,681, LR chi2(10) = 7610.52, Prob > chi2 = 0.0000, Log likelihood = -49475.531, Pseudo ^R^2 = 0.0714

**Table 2:** Predictive accuracy of a model of hookworm prevalence in Africa*.

Validation statistic	Prevalence threshold	
	
	5%	50%
AUC	0.76	0.70
Optimal threshold	0.31	0.36
Sensitivity (%)	78.6	67.7
Specificity (%)	79.7	68.5
PPV (%)	88.7	50.8
NPV (%)	68.9	81.5

*Validation statistics including area under the curve (AUC), optimal prediction threshold and sensitivity, specificity, positive predictive value (PPV), and negative predictive value (NPV) at the optimal prediction threshold are presented for observed prevalence thresholds of 5% and 50%.

The spatial distribution of four principle malaria transmission settings, adjusted for urbanisation, indicates that malaria risk is spatially discrete in Botswana, Namibia and South Africa, and for the remainder of SSA, the majority of the population is exposed to stable malaria transmission (Figure 1b). Throughout SSA, 90.8 million (50.7%) school-aged children are exposed to stable endemic malaria transmission (FCS Class 3). In these areas, 32.1 (95% CI: 31.4–32.7) million (17.9% of total population) children aged 5–14 years are estimated to be at risk of co-infection with *P. falciparum *and hookworm. It is also estimated that 9.6 (95% CI: 9.3–9.8) million children are at risk of coincident infection in acute seasonal and 3.4 (95% CI: 3.2–3.5) million in areas of marginal malaria infection risk. In total, therefore, 45.1 million (95% CI: 43.9–46) (25%) school-aged children in SSA are at coincidental risk of hookworm and malaria infection risk. With the exception of southern Africa and the Horn of Africa, high transmission levels of both hookworm and *P. falciparum *occur throughout much of SSA. Only in the Sahelian areas of west and central Africa does stable malaria occur without hookworm and only in parts of southern Africa does hookworm occur without malaria (Figure 1c).

## Discussion

This paper provides detailed information on both the predicted distribution of hookworm infection among African school-aged children and the possible co-distribution of hookworm and *P. falciparum *malaria. The developed model estimates that throughout Africa, hookworm is widespread and that over a quarter of school-aged children appear to be at risk of co-infection with malaria and hookworm. These estimates are based on uniquely detailed epidemiological data available from dedicated school-based surveys and from a comprehensive search of the published literature, and used in combination with satellite-derived environmental data and high-resolution human population distribution maps. The results provide an empirical basis for defining future disease burden estimates for parasitic diseases among African school children, and help identify future priority areas for research and policy investment.

It has recently been speculated that infection with helminths, including hookworm, may increase susceptibility to clinical malaria [7-9]. This hypothesis is based on immunological models which suggest that helminth infections are associated with chronic immune activation which affect the acquisition of immunity to malaria. More specifically, helminth infections tend to promote a type 2 bias in the immune response, involving the production of the cytokines interleukin-4 (IL-4), IL-5, IL-10, and IL-13, as well as immunoglobulin E [29]. This bias can affect the production of non-cytophilic, clinically ineffective, antibodies and hence, makes individuals more susceptible to clinical malaria. However, the hypothesis has not yet been substantiated by robust clinical data and the limited observational data that do exist have yielded contradictory findings [reviewed in [9]].

Another potential consequence of co-infection with malaria and hookworm is increased risk of anaemia. Malaria contributes to reduce haemoglobin concentrations through a number of mechanisms, principally by increasing rates of destruction and removal of parasitized and non-parasitized red cells and decreasing the rate of erythrocyte production in the bone marrow. Some of the mechanisms that cause anaemia during malaria are associated more with the acute clinical states (e.g. hemolysis or cytokine disturbances), whereas chronic or repeated infections are more likely to involve dyserythropoiesis [30]. By contrast, hookworm causes anaemia through the process of intestinal blood loss [4] and the degree of pathology is related to the intensity of worm infection [31]. Given the distinct mechanisms by which *P. falciparum *and hookworm reduce haemoglobin concentrations, it is probable that malaria and hookworm would be additive in their ability to cause anaemia. It is also possible that women of child-bearing age are likely to be vulnerable to anaemia associated with co-infection.

Surprisingly, there remains a paucity of epidemiological investigation on the health consequences of co-infection with *P. falciparum *and hookworm. This makes it difficult to articulate the cumulative public health consequences of co-infection in an African setting. Nevertheless, it is probable that infection with *P. falciparum *or hookworm during a child's school years disadvantages the child and co-infection is not only worse for the child but co-distribution might provide an opportunity for dual school-based control.

There have been recent proposals to coordinate the control of parasite diseases between agencies traditionally focused on single pathogens [10,11,33]. At the same time, it is recognized that coverage of interventions to prevent anaemia is poor and that a non-pathogen specific approach is required if the burden of anaemia in SSA is to be reduced [32]. There exists already a coordinated focus for helminth control with the combined delivery of praziquantel to treat schistosomiasis and benzimidazole anthelmintics, albendazole and mebendazole, to treatment hookworm and other soil-transmitted helminth infections. The focus of control efforts is the school age population because the most intense worm infections and related illnesses occur at school age and infection can have adverse consequences for health and development, many of which is rapidly reversed by treatment. For these reasons, school age children are the natural targets for treatment, and school based treatment delivery programmes offer major cost advantages because of the use of the existing school infrastructure and the fact that schoolchildren are accessible through schools [33].

In terms of malaria control, the delivery of malaria chemoprophylaxis to schoolchildren through schools was widespread in Africa during the 1950's and 1960's, and resulted in reductions in parasitaemia greater than 75% [34]. Significant improvements were also seen in mean haemoglobin levels, rates of severe anaemia and clinical malaria attacks. However, regular chemoprophylaxis in malaria-endemic countries proved to be unsustainable, largely due to problems in drug distribution and financing, and to concerns about the emergence of drug resistance. Recently, however, the effectiveness of teachers in recognizing and treating malaria in school children has been demonstrated in Tanzania [35]. The programmatic use of presumptive treatment has been evaluated in Malawi [36] where teachers were trained to use "first aid kits" to dispense sulfadoxine-pyrimethamine tablets to affected children, following national guidelines. There is, however, an absence of consistent guidance as to good practice for malaria control in schools [37,38].

The observed statistical relationships between hookworm prevalence and large-scale environmental data are consistent and interpretable with the known biology of hookworm. Experimental studies indicate that hookworm larvae present in the environment develop and die at temperature-dependent rates. For instance, maximum survival rates of hookworm larvae, as indicated by proportion of larvae surviving, occur at 20–30°C and development of hookworm larvae ceases at 40°C [39]. In addition, field studies show that the abundance of hookworm larvae is related to atmospheric humidity [40]. Differences in vegetation, as indicated by NDVI, may be a useful proxy for soil moisture and humidity, since a large amount of vegetation tends to prevent evaporation and conserve soil moisture. Given the importance of environmental factors on transmission processes, it is unsurprising that statistical relationships between large-scale environmental factors and spatial patterns of infection can be observed.

It has previously been suggested that hookworm is a rural disease [41]. However, comparable data on hookworm infection in urban and rural settings are remarkably few and those that do exist indicate that hookworm appears to be equally prevalent in both urban and rural settings [reviewed in [16]]. This was confirmed by our initial analysis which indicated that the degree of urbanization was not associated with patterns of hookworm infection. Malaria transmission, on the other hand, is lower in urban areas compared to rural areas, due to a variety of factors including greater wealth and access to treatment, mosquito avoidance behaviour by urban populations, pollution of potential larval habitats, and higher human population densities which may reduce biting rates [26,28].

At local scales other factors including variability in sanitation and socio-economic status have to be considered. However, the precise extent to which socio-economic status [SES] is associated with hookworm or malaria infection in Africa is not clear, with studies yielding contrasting results [42,43]. Such apparent contradiction can be reconciled by the fact that hookworm occurs in the poor regions of Africa and insufficient variation in SES exists among individual populations for significant associations to occur. Moreover, few detailed SES data exist at sufficiently fine spatial scale. These features make it difficult to incorporate socio-economic factors into our model.

The approach adopted in the present analysis highlights a number of key methodological limitations. First, the errors surrounding our estimates of populations at risk only take into account those arising from fitting of the logistic regression models to the observed hookworm data and, therefore, underestimate the true error of the population-at-risk estimates by an unknown quantity. This is because there are a number of other potential sources of error that are not included in our estimates, ranging from measurement error of parasitological diagnosis and the satellite-derived independent variables, to multiple sources of error in the population density maps. Additionally, as the current models did not incorporate spatial correlation, a known feature of parasitological data, this will have led to further underestimation of true error. Second, there are limitations with the MARA malaria risk map. While the MARA model has enjoyed wide use, it has been validated against parasite prevalence data only in Kenya [44]. Extensive, contemporary, reliable and validated estimates of *P. falciparum *malaria transmission intensity across Africa are lacking despite long advocacy for their importance [45]. Revised models of malaria intensity are currently being developed based on parasitological data for Africa and regions outside of Africa as part of the Malaria Atlas Project [46]. Third, there are also limits on the present ability to define urban populations. Research is ongoing to improve population [47] and urban extent delineations [48] in Africa. Over the next few years improved approaches to mapping infectious disease, population distributions and settlement patterns will iteratively improve our estimations of age-stratified and projected denominators for multiple disease burden analyses and geographical targeting of interventions.

Finally, the extent to which co-distribution across population implies co-infection within individuals cannot accurately be defined on the basis of the current data. Nevertheless, the occurrence of co-infection is probably higher than simple probability would suggest due to the observed clustering of both infections in certain individuals and households [49,50]. This means that the current estimates may, in fact, be too low. Given the demonstrated widespread spatial congruence of both infections, better descriptions of within population distributions and risks of co-infection are both clearly needed to better define the contribution of co-infection to overall disease burden.

## Conclusion

The present analysis shows that malaria and hookworm infection are widespread throughout SSA and over a quarter of school-aged children in sub-Saharan Africa appear to be at risk of coincident infection and thus at enhanced risk of clinical disease. The results highlight an important research agenda that includes investigating both the causation and consequences of co-infection with *P. falciparum *and hookworm. In terms of school health programmes, they also suggest that the control of parasitic helminths and of malaria in school children, interventions which tend currently to be perceived as having separate goals, could in fact be viewed as essential co-contributors to promoting the health of children.

## Authors' contributions

The study was conceived by SB with input from RWS. SB collated the survey data and ACAC developed the statistical models. SIH provided the satellite data and the adjusted malaria risk model, and AJT undertook geographical extractions and analyses of these data. PJH, DAPB and RWS provided epidemiological interpretation of the data and background material. SB wrote the first draft of the paper and all authors contributed to, read and approved the final manuscript.

## Supplementary Material

Additional File 1 Data used to develop predictive models of hookworm. The prevalence data used to develop the predictive models of hookworm, including data sources and sample sizes. Click here for file
