# Supplementary material for: The co-distribution of Plasmodium falciparum and hookworm among African schoolchildren
Source: Malar J. 2006 Nov 3;5:99. doi: 10.1186/1475-2875-5-99 (PMC1635726; doi:10.1186/1475-2875-5-99)
Supplement: Additional File 1 — Data used to develop predictive models of hookworm. The prevalence data used to develop the predictive models of hookworm, including data sources and sample sizes. [file 1475-2875-5-99-S1.doc]

Additional file 1: Data used to develop hookworm models.

The prevalence data used in the analysis are shown in the table below. Only data for pre-intervention populations were used. In all studies only schoolchildren were randomly sampled and presence of infection was based on parasitological diagnosis, mainly the Kato-Katz method. The latitude and longitude of each school was obtained from either a hand-held global positioning system used during the survey or detailed ordinance survey maps.

Table. Data on the prevalence of infection used to develop predictive models of hookworm

| Country | Region | Number of schools | Number of children | Ref |
| --- | --- | --- | --- | --- |
| Botswana | Kweneng | 1 | 302 | 1 |
| Burkina Faso | Bazega | 8 | 761 | 2 |
| Cameroon | Countrywide | 401 | 18,198 | 3 |
| Chad | Countrywide | 20 | 1,023 | 4 |
| Eritrea | Countrywide | 40 | 1,607 | 5 |
| Gambia | Western | 1 | 128 | 6 |
| Guinea | Countrywide | 18 | 2,754 | 7 |
| Kenya | Western | 48 | 3,320 | 8 |
| Kenya | Countrywide | 21 | 2,235 | 9 |
| Kenya | Western | 7 | 1,235 | 10 |
| Mali | Sikasso | 5 | 392 | 11 |
| Malawi | Chiradzulu | 1 | 280 | 12 |
| Malawi | Karonga | 4 | 477 | 13 |
| Mauritania | Trarza | 2 | 195 | 14 |
| Mozambique | Marromeu | 1 | 60 | 15 |
| Mozambique | Xai-Xai | 10 | 503 | 15 |
| Mozambique | Bilene | 10 | 532 | 15 |
| Namibia | Caprivi | 6 | 367 | 16 |
| South Africa | Kwa-Zulu Natal | 10 | 1,561 | 17 |
| South Africa | Kwa-Zulu Natal | 9 | 681 | J. Kvalsvig, unpublished data |
| South Africa | Kwa-Zulu Natal | 5 | 586 | 18 |
| South Africa | Kwa-Zulu Natal | 16 | 1,566 | C Appleton,  unpublished data |
| South Africa | Kwa-Zulu Natal | 5 | 592 | 19 |
| Tanzania | Mwanza | 54 | 6,332 | 20 |
| Tanzania | Kagera, Mwanza, Tabora, Shinyanga | 143 | 8,621 | 21 |
| Tanzania | Tanga | 38 | 1,827 | 22 |
| Uganda | Countrywide | 171 | 10,445 | 23 |
| Zambia | Southern | 58 | 3,470 | 24 |
| Zambia | Eastern | 31 | 1,631 | PCD, unpublished data |

References

1. Michaelsen K F (1985) Hookworm infection in Kweneng District, Botswana. A prevalence survey and a controlled treatment trial. Trans Roy Soc Trop Med Hyg 79: 848-851.
2. Sacko M, Maiga F, Roschnik N, Sobgo G (2000) Etudes et interventions menees par sc-usa/fdc dans la zone de Sapone au Burkina Faso. Ouagadougou: Save the Children.
3. Ratard RC, Kouemeni LE, Ekani Bessala MK, Ndamkou CN (1988) Distribution of hookworm infection in Cameroon. Ann Trop Med & Parasitol 86: 413-418.
4. Brooker S, Beasley NMR, Ndinaromtan M, Madjiouroum EM, Baboguel M et al. (2002) Use of remote sensing and a geographical information system in a national helminth control programme in Chad. Bull World Health Organ 80: 783-789.
5. Partnership for Child Development (2003) A situation analysis of the health of school children in Eritrea. London: Imperial College.
6. Palmer D R, Bundy DAP (1995) Epidemiology of human hookworm and Ascaris lumbricoides infestations in rural Gambia. East Afr Med J 72: 527-530.
7. Gyorkos T W, Camara B, Kokoskin E, Carabin H, Prouty R (1996) Survey of parasitic prevalence in school-aged children in Guinea. Sante 6: 377-381.
8. Brooker S, Miguel EA, Moulin S, Waswa P, Namunyu R, Guyatt H & Bundy DAP. (2001). The potential of rapid screening methods for *Schistosoma mansoni* in Western Kenya. Ann Trop Med Parasitol 95: 343-351.
9. Mwaniki D, Omwenga A, Muniu E, Mutunga J, Akelola R et al. (2002) Anaemia and status of iron, vitamin A and zinc in Kenya: The 1999 national survey report. Nairobi: UNICEF.
10. Clarke S, Brooker S, Njagi K, Njau E, Estambale B, Muchiri E & Magnussen P. (2004). Malaria morbidity amongst schoolchildren living in two areas of contrasting transmission in western Kenya. Am J Trop Med Hyg 71, 732-738.
11. Sacko M, Roschnik N, Maiga I, Gorsline E (1999) Assessment of the health and nutritional status of school children in 10 community schools in Kolondieba, Mali: report of the school health and nutrition initiative’s preliminary survey. Bamako: Save the Children.
12. Phiri K, Whitty CJ, Graham SM, Ssembatya-Lule G (2000) Urban/rural differences in prevalence and risk factors for intestinal helminth infection in southern Malawi. Ann Trop Med & Parasitol 94: 381-387.
13. Randall AE, Perez AM, Floyd S, Black GF, Crampin A et al. (2002) Patterns of helminth infection and relationship to BCG vaccination in Karonga District, northern Malawi. Trans R Soc Trop Med Hyg 96: 29-33.
14. Urbani C, Toure A, Hamed AO, Albonico M, Kane I et al. (1997) Parasitoses intestinales et schistosomiases dans la vallee du fleuve Senegal en Republique Isamique de Mauritanie. Med Tropicale 57: 157-160.
15. Bobrow EA, Zacher AM (1999) School health and micronutrient: a baseline report for Xai-Xai and Bilene districts in Gaza Province, Mozambique. Maputo: Save the Children.
16. Schutte CHJ, Van Deventer J (1990) Schistosomiasis in eastern Caprivi. Part I. The prevalence of Schistosoma species and other parasitic infections in schoolchildren. Durban: Medical Research Council.
17. Appleton CC, Maurihungirire M, Gouws E (1999) The distribution of helminth infections along the coastal plain of Kwazulu-Natal province, South Africa. Ann Trop Med Parasitol. 93: 859-868.
18. Appleton CC, Gouws E (1996) The distribution of common intestinal nematodes along an altitudinal transect in Kwa-Zulu Natal, South Africa. Ann Trop Med Parasitol 90: 181-188.
19. Saathoff E (2002) Geohelminth and Schistosoma haematobium infection in schoolchildren from rural northern KwaZulu-Natal/South Africa. PhD Thesis. Charlottenlund and Copenhagen: Danish Bilarziasis Laboratory and University of Copenhagen.
20. Lwambo NJS, Siza JE, Brooker S, Bundy DAP, Guyatt H (1999) Patterns of concurrent infection with hookworm and schistosomiasis in school children in Tanzania. Trans R Soc Trop Med Hyg 93: 497-502.
21. Clements ACA, Lwambo NJS, Blair L, Nyandindi U et al. (2006). Bayesian spatial analysis and disease mapping: tools to enhance planning and implementation of a schistosomiasis control programme in Tanzania. Trop Med Int Hlth (in press)
22. Bundy DAP, Brooker S, Hall A, Adjei S, Allen E, Amuah E et al. (1998) The health of school-age children: experience from school health programmes in Ghana and Tanzania. Trans R Soc Trop Med Hyg 92: 254-261.
23. Kabatereine NB, Tukahebwa EM, Kazibwe F, Twa-Twa JM, Barenzi JFZ et al. (2005) Soil-transmitted helminthiasis in Uganda: epidemiology and cost of control. Trop Med Int Hlth 10: 1187-1189.
